# Phylogeny of *Anopheles* (*Kerteszia*) (Diptera: Culicidae) Using Mitochondrial Genes

**DOI:** 10.3390/insects11050324

**Published:** 2020-05-24

**Authors:** Karin Kirchgatter, Lilian de Oliveira Guimarães, Henrry Hugo Yañez Trujillano, Fernando Rafael Arias, Abraham Germán Cáceres, Ana Maria Ribeiro de Castro Duarte, Rosely dos Santos Malafronte, Rosa Maria Tubaki, Maria Anice Mureb Sallum

**Affiliations:** 1 Laboratório de Bioquímica e Biologia Molecular, Superintendência de Controle de Endemias/Instituto de Medicina Tropical de São Paulo, Universidade de São Paulo, SP 01027-000, Brazil; lilianguima@gmail.com (L.d.O.G.); amrcd2@gmail.com (A.M.R.d.C.D.); 2 Area de Vigilancia y Control Vectorial, Dirección de Salud Ambiental, Dirección Ejecutiva de Salud Ambiental, Dirección Regional de Salud Cusco, Cusco 08003, Peru; henrryhugo@yahoo.com; 3 Laboratorio de Referencia Regional de Salud Pública, Dirección Regional de Salud Puno, Puno 21002, Peru; fariasvaldivia@hotmail.com; 4 Seccion de Entomología, Instituto de Medicina Tropical “Daniel A. Carrión” y Departamento Académico de Microbiología Médica, Facultad de Medicina, Universidad Nacional Mayor de San Marcos, Lima 15081, Peru; acaceres31@hotmail.com; 5 Laboratorio de Entomologia, Instituto Nacional de Salud, Lima 15064, Peru; 6 Laboratório de Protozoologia, Instituto de Medicina Tropical de São Paulo, Universidade de São Paulo, São Paulo, SP 05403-000, Brazil; rmalafronte@usp.br; 7 Laboratório de Entomologia Médica, Superintendência de Controle de Endemias, São Paulo, SP 01027-000, Brazil; tubaki.rm@gmail.com; 8 Departamento de Epidemiologia, Faculdade de Saúde Pública, Universidade de São Paulo, São Paulo, SP 01246-904, Brazil; masallum@usp.br

**Keywords:** mosquitoes, malaria, *Kerteszia*, Peru, population genetics, barcoding

## Abstract

Identification of mosquito species is necessary for determining the entomological components of malaria transmission, but it can be difficult in morphologically similar species. DNA sequences are largely used as an additional tool for species recognition, including those that belong to species complexes. *Kerteszia* mosquitoes are vectors of human and simian malaria in the Neotropical Region, but there are few DNA sequences of *Kerteszia* species in public databases. In order to provide relevant information about diversity and improve knowledge in taxonomy of *Kerteszia* species in Peru, we sequenced part of the mitochondrial genome, including the cytochrome c oxidase I (COI) barcode region. Phylogenetic analyses structured all species of mosquitoes collected in Peru into a single clade, separate from the Brazilian species. The Peruvian clade was composed of two lineages, encompassing sequences from *Anopheles (Kerteszia) boliviensis* and *Anopheles (Kerteszia) pholidotus*. *An. pholidotus* sequences were recorded for the first time in Peru, whereas *An. boliviensis* sequences were for the first time published in the GenBank database. Sequences generated from specimens morphologically identified as *Anopheles (Kerteszia) cruzii* clustered into three separate clades according to the collection localities of Serra do Mar, Serra da Mantiqueira, and Serra da Cantareira, confirming *An. cruzii* as a species complex, composed of at least three putative species.

## 1. Introduction

Malaria is a mosquito-borne tropical disease that remains an important public health problem in some tropical and subtropical countries. In South America, the majority of cases occur in areas of the Amazon rainforest, and are mainly concentrated in Venezuela, Brazil, and Peru, which registered 30%, 24%, and 19% of the American malaria cases in 2015, respectively [1].

In Peru, malaria transmission occurs throughout the year in rural areas below 2000 m altitude, with the highest risk in the department of Loreto in the Amazon Region, which registered 96% of the 55,218 cases reported in the country in 2017 [2]. In 2017, the department of Cusco, where mosquitoes from this study were collected, registered an increase of 41% in the malaria cases in relation to 2016, being the fifth department in Peru in number of cases [2]. In Lima and areas at high altitude (including the Inca Trail, Machu Picchu, and Lake Titicaca) the malaria risk is low. In 2019, there was a decrease in cases of malaria, with 56% fewer reported cases compared to the same period in 2018. However, an outbreak of 34 cases of malaria due to *Plasmodium vivax* was reported in Tumbes Department, in the northern part of the country, which included 22 autochthonous cases and 12 cases imported from Venezuela; this outbreak highlights the risk of re-introduction of malaria in a historically endemic area where transmission had been interrupted in recent years [3].

*Plasmodium vivax* is responsible for 80% of the Peruvian malaria cases [4]. However, as in other endemic countries, the incidence of *Plasmodium malariae* may be underestimated due to difficulties in microscopic differentiation from *P. vivax*, and its presence in coinfections. *Plasmodium malariae* has been identified in the vast majority of blood samples from Amerindian ethnic groups from Peru, including coinfection with *P. vivax* [5]. In areas of the Peruvian Amazon, the primary vector is *Anopheles darlingi*. This species accounts for >85% of the anopheline mosquitoes that were observed to be blood-feeding on humans, and it is responsible for malaria transmission, particularly in frontier areas [6,7,8,9]. In Iquitos, Peruvian Amazon, a high proportion of *An. darlingi* was found blood-feeding on humans and avians, showing the vector a generalist mosquito [10].

In Brazil, 99% of malaria cases registered are from the Amazon Region, where *An. darlingi* is the primary vector, and other species can participate as secondary vectors in specific areas [11]. Outside the Amazon region, malaria endemic areas are mainly related to the Atlantic forest biome where *Kerteszia* species are associated with phytotelma habitats, such as those in the Bromeliaceae family, where they use the axils as a larval habitat [12]. In the Atlantic Forest, *Plasmodium* infection is usually asymptomatic, caused by sub-patent levels of *P. vivax* or *P. malariae* [13], and *Anopheles (Kerteszia) cruzii* and *Anopheles (Kerteszia) bellator* are their vectors, with *An*. *cruzii* involved in the transmission of both human and simian malaria [14,15,16,17].

*Anopheles* species that use bromeliads phytotelmata as larval habitats have been recognized as a distinct group by Knab (1913). The relationship between the subgenera *Kerteszia* and *Nyssorhynchus* has been hypothesized by Root (1922). Following this, Christophers (1924) and Edwards (1932) included *Kerteszia* in the subgenus *Nyssorhynchus*, but Dyar (1928) and Komp (1937) treat them as a distinct subgenera [18]. Recently, Foster et al. [19] proposed *Kertezia* as a genus on the basis of the robust results of mitogenome phylogeny of the subfamily Anophelinae. There are 12 valid *Kerteszia* species: *Anopheles (Kerteszia) auyantepuiensis*, *Anopheles (Kerteszia) bambusicolus*, *Anopheles (Kerteszia) bellator*, *Anopheles (Kerteszia) boliviensis*, *Anopheles (Kerteszia) cruzii*, *Anopheles (Kerteszia) gonzalezrinconesi*, *Anopheles (Kerteszia) homunculus*, *Anopheles (Kerteszia) laneanus*, *Anopheles (Kerteszia) lepidotus*, *Anopheles (Kerteszia) neivai*, *Anopheles (Kerteszia) pholidotus*, and *Anopheles (Kerteszia) rollai* [20].

*Kerteszia* mosquitoes are widely distributed from Mexico to southern Brazil [18,21,22]. In Peru, there are records of *An*. *bambusicolus*, *An. boliviensis*, *An. cruzii*, *An. homunculus*, *An. laneanus*, *An. lepidotus*, *An. neivai*, and *An. pholidotus* (ref. in [17]). In Brazil, the occurrences of *An. bambusicolus* (in areas of the interior of the Atlantic Forest), *An. bellator*, *An. cruzii* and *An. homunculus* (in coastal and mountainous areas of the Serra do Mar in the Atlantic Forest Region), *An. laneanus* (in areas of Serra da Mantiqueira), and *An. neivai* (in the Amazon Region) have been registered (ref. in [17]). *An. boliviensis* was registered in Brazil [23] but the absence of recent records makes its identification questionable [20].

*Anopheles homunculus*, *An. cruzii*, *An. bellator*, and *An. laneanus* form a well-defined species group, which is always easily separated from the other *Kerteszia* species [18]. *Anopheles homunculus* and *An. cruzii* are morphologically similar on the basis of the external characteristics of the females. However, they can be easily identified by characteristics of male genitalia, as well as fourth-instar larva and pupa [24,25]. *Anopheles cruzii* has been demonstrated to be a species complex on the basis of cytological differences on the banding patterns of polythene chromosomes [26]. Recently, Oliveira et al. [27], using the mitogenome, showed that the specimens from Serra da Cantareira, São Paulo, Brazil, may belong to distinct lineage that is sister to *An. cruzii*.

Differences in the ecology and behavior of species can affect the transmission of malaria and the success of the methods used in the control, such as insecticides and insecticide-impregnated bed nets [28]. Therefore, the characterization of species composition of a local population for the design of vector control projects is of vital importance. DNA-based methods have been widely used for species identification. Mitochondrial genes have been broadly employed in studies focusing on taxonomy and phylogeny of the Culicidae and Anophelinae subfamilies. The cytochrome c oxidase subunit I (*COI*) gene of the mitogenome has been largely used to identify taxonomic groups at species level [29,30]. There are COI sequences available in the GenBank and BOLD databases, including sequences generated for the Mosquito Barcoding Initiative (MBI) (http://barcodeoflife.ning.com/group/mosquitobarcoding).

In the current study, we initially used in the phylogenetic analysis a fragment of mitochondrial cytochrome c oxidase I (COI) that represents the barcode region [31,32]. The aim was to analyze the haplotype diversity and verify the genetic variability of specimens collected in Peru. This mitochondrial region is the main source of reference sequences, and has been successfully used to identify mosquitoes [25,29,33,34] and to evaluate intraspecific variability, especially in Neotropical *Anopheles* [35,36,37]. This analysis included mosquitoes from Peru, which were previously identified as *An. cruzii*, *An. boliviensis*, *An. Laneanus*, and *An. lepidotus* using morphological characteristics. Lastly, we generated two more partial sequences (another *COI* region and *ND4*, NADH dehydrogenase subunit 4 gene), totalizing 10% of the whole mtDNA (mitochondrial DNA) (≈1500 bp), and compared with reference sequences from a previously published complete mitochondrial DNA genome, to verify whether *An. cruzii* is a species complex.

## 2. Materials and Methods

### 2.1. Mosquito Sampling and Handling

Females were collected in different localities and dates (Table 1 and Table 2). In Brazil, mosquitoes were collected using CDC (Centers for Disease Control) light traps with CO_2_ (dry ice) for 12 h, beginning at twilight, or with Shannon trap for 3 h, from 6:00 p.m. to 9:00 p.m. Peruvian mosquitoes were collected with Shannon traps, from 6:00 p.m. to 9:00 p.m. Mosquitoes were killed with chloroform vapor and transported to the laboratory for identification, using morphological taxonomy key by Zavortink [18], Consoli and Lourenço-de-Oliveira [38], and Forattini [12]. Mosquitoes were individually stored at −20 °C in 1.5 mL plastic tubes sealed with parafilm.

### 2.2. Genomic DNA Extraction and PCR Amplification of Mitochondrial Gene Fragments

Mosquitoes were crushed with disposable tips in lysis buffer, and genomic DNA (gDNA) was obtained using PureLink Genomic DNA Purification Kit (Invitrogen), according to the manufacturer’s instructions.

A fragment of 710 base pairs (bp) of the barcode region of the mitochondrial *COI* gene was generated by PCR amplification using the primer pair LCO1490 and HCO2198 [39], following the protocol proposed by Ruiz et al. [40]. A fragment of ≈600 bp of COI gene (gene region used in phylogeny) was obtained as described [41] with the primers COIF (5′-GGA TTA TTA GGA TTT ATT GT-3′) and COIR (5′-GCA AAT AAT GAA ATT GTT CT-3′). A fragment of ≈400 bp of the mitochondrial *ND4* gene was generated using the primers ND4+ (5′-GTD YAT TTA TGA TTR CCT AA-3′) and ND4-(5′-CTT CGD CTT CCW ADW CGT TC-3′) and the PCR program, as described [42].

### 2.3. Sequencing, Alignment, and Phylogenetic Analysis

Amplified fragments were sequenced directly in both directions by BigDye Terminator v3.0 Cycle Sequencing Kit in ABI Genetic Analyzer (Applied Biosystems^®^, Foster City, CA, USA), using the corresponding flanking primers. The COI and ND4 sequences obtained from newly sequenced mosquitoes were deposited in the GenBank database (COI: MH589352-MH589421; ND4: MH560305-MH560342). Sequences were aligned with the reference sequences (Table 3) using Clustal X version 1.81 [43]. Alignments were then inspected and edited in MEGA version 5.1 [44].

Two methods of phylogenetic reconstruction were implemented using the Bayesian approach. In the first round of analysis, 624 bp of the *COI* barcoding region was employed, due the higher availability of sequences of this *COI* gene region for the *Anopheles* diversity in the GenBank database, from which all the sequence data of reference *Kerteszia* species were downloaded. In the second round of analysis, phylogenetic relationship was investigated using *ND4* and *COI* genes. *COI* and *ND4* datasets were concatenated, after trimming the primers off from the sequences—including 338 bp of the *ND4* gene, 511 bp of the *COI*, and 624 bp of the barcode region of the COI, generating a total of 1473 bp. In this analysis only reference sequences from complete mtDNA were used, avoiding an incomplete final matrix and missing data. All sequence accession numbers used in both analyses are provided in Table 1, Table 2 and Table 3 and added into phylogenetic trees.

The phylogenetic reconstructions were performed using the Bayesian approach implemented in MrBayes v3.2.0 [45]. Bayesian inferences were run with two Markov Chain Monte Carlo searches of 3 million generations, each with sampling of 1 in 300 trees. After a burn-in of 25%, the remaining 15,002 trees were used to generate a 50% majority-rule consensus tree. The results of analyses were visualized using FigTree version 1.4.0 [46]. The topologies were rooted using sequences obtained for species of the subgenera *Anopheles* or *Nyssorhynchus* (Table 2 and Table 3).

## 3. Results

**Figure 1:**
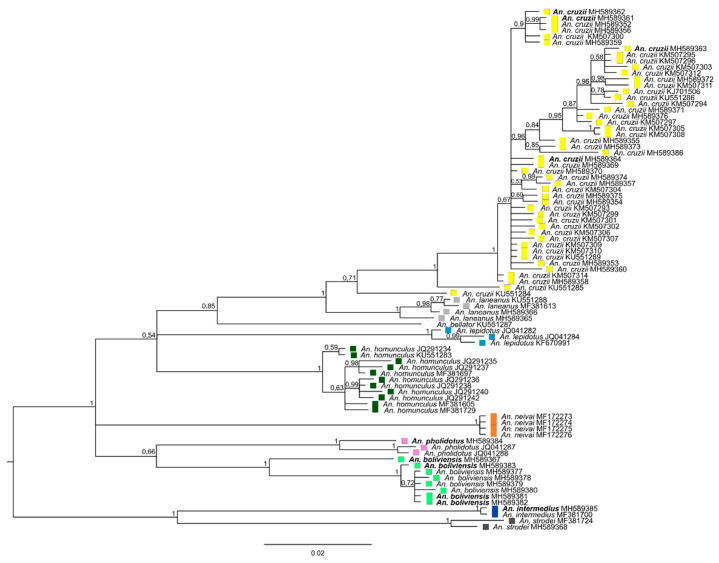
Phylogeny of the *Kerteszia* species using cytochrome c oxidase I (COI) barcoding region by Bayesian inference tree. Posterior probability values are shown to each clade. Yellow, *An. cruzii* clade; grey, *An. laneanus* clade; white, *An. bellator*; blue, *An. lepidotus*; dark green, *An. homunculus*; orange, *An. neivai*; pink, *An. pholidotus*; green, *An. boliviensis*; dark blue, *An. (Anopheles)* species; dark grey, *An. (Nyssorhynchus)* species. Specimens reclassified are highlighted in bold (see Table 1 and Table 2).

The results of the analyses using only DNA sequences of the COI barcoding region are shown in Figure 1. This approach was used with the aim of increasing the number of sequences in the phylogenetic tree, as there are no complete mitochondrial sequences available for some *Kerteszia* species such as *An. neivai*, *An*. *pholidotus,* and *An*. *lepidotus.* The sequence alignment composed of 85 sequences with 624 bp length—9 from Peru, 26 from Brazil, and 50 references from GenBank—had 168 variable sites. The Bayesian phylogenetic tree (Figure 1) shows that the sequences from nine specimens from Peru grouped together in a strongly supported clade. The Peruvian species clade contains two major groups. One group encompasses sequences from specimens identified as *An. boliviensis*, *An. cruzii*, *An. laneanus*, and *An. lepidotus*. The second group contains sequences from *An. lepidotus* that clustered with *An. pholidotus* from Venezuela.

**Figure 2:**
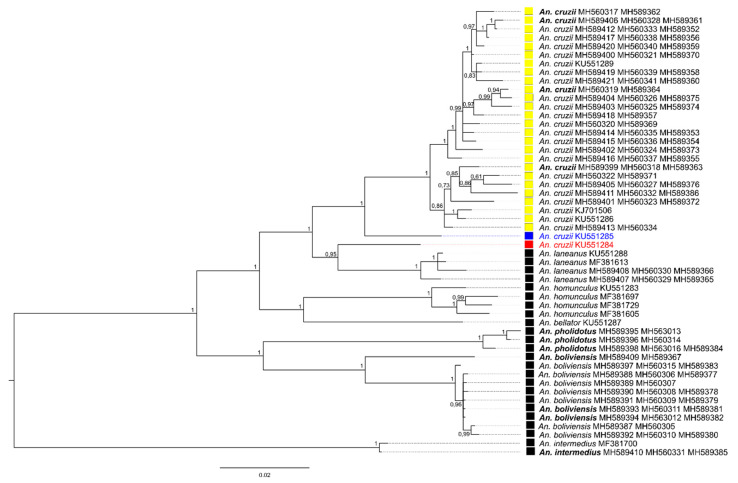
Phylogeny of the *Kerteszia* species using COI and NADH dehydrogenase subunit 4 (ND4) genes by Bayesian inference tree. Posterior probability values are shown to each clade. *An. cruzii* from is Serra da Cantareira represented in red. *An. cruzii* from Serra da Mantiqueira is represented in blue. *An. cruzii* from Serra do Mar is represented as a yellow box. Specimens reclassified are highlighted in bold (see Table 1 and Table 2).

**Figure 3:**
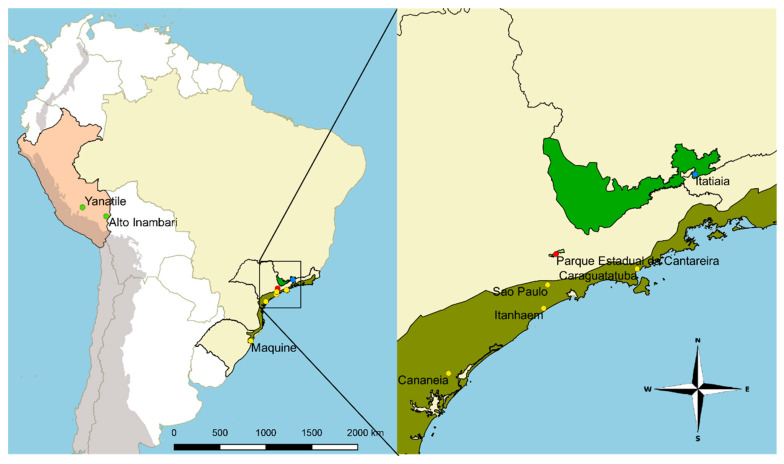
(**a**) Origin localities map for *An. cruzii* sequences from Peru and Brazil used in this study. (**b**) Localities of Itatiaia/RJ (Serra da Mantiqueira, blue dot); Parque Estadual da Cantareira/SP (Serra da Cantareira, red dot); Cananéia/SP, Caraguatatuba/SP, Itanhaém/SP, and São Paulo/SP (all in Serra do Mar, yellow dots) are shown.

For the second round of Bayesian analysis, approximately 1.5 kb (10% of the whole mtDNA) was employed, including COI and ND4 genes of 39 specimens of *An.* (*Kerteszia*) species from Peru and Brazil. Twelve new sequences were blasted with sequences available in GenBank for other *Kerteszia* species to verify identification and potential presence of stop codon in each sequence. The alignment generated 1473 bp with 371 variable sites. Sequences of *Anopheles* (*Anopheles*) species were used as outgroup. All clades recovered in the Bayesian phylogenetic analysis were strongly supported (Bayesian Posterior Probability—BPP = 1). All the *Anopheles* (*Kerteszia*) species were grouped in a clade that was separated into two clades: one containing all Peruvian species, and the second clade being formed of species collected in Brazil (Figure 2). The Peruvian species clade was split in two minor groups. Species from Brazil were separated in two clades: one clade composed of *An. bellator* and *An. homunculus*, and a second clade composed of *An. laneanus* and *An. cruzii*, with four mosquitoes morphologically identified as *An. bellator* (one of which was collected in 1946). Sequences of *An. cruzii* from Brazil were separated into clades in accordance with the geographical localities of Serra do Mar, Serra da Mantiqueira and Serra da Cantareira (Figure 2 and Figure 3).

## 4. Discussion

Recently, the number of publications using mitochondrial sequences for inferring phylogenetic relationships within the *Kerteszia* has increased [47,48,49,50,51,52,53]. However, there are few studies focusing on populations of *An. cruzii* [19,27,54], with no publication regarding *Kerteszia* species that occur in Peru. In order to provide relevant information about diversity and improve knowledge in taxonomy of *Kerteszia* species from Peru, we sequenced two mitochondrial genes (COI and ND4), including the DNA barcode region.

The analysis of genetic differentiation of COI and ND4 genes indicated that there are at least two *Kerteszia* species of that occur in Peru. Preliminary identification of specimens of *An. cruzii* and *An. lepidotus* using morphological characters of the female was not confirmed by phylogenetic analyses of mitochondrial genes. In fact, specimens from Peru that were identified as *An. cruzii* had scales on the abdominal terga, indicating that they belonged to other species. In the Bayesian topology, those specimens of *An. cruzii* with scales on the abdomen terga grouped with most of the mosquitoes from Peru, which were identified as *An. boliviensis*. In addition, one specimen morphologically identified as *An. lepidotus* clustered with *An. pholidotus*, with an identity of 98% in the COI barcoding region. The clade encompassing *An. pholidotus* sequences was recovered and was well separated from *An. lepidotus*. Moreover, a Peruvian specimen preliminary identified as *An. laneanus* clustered with *An. boliviensis*. For accurate identification of *An. boliviensis*, *An. pholidotus*, and *An. lepidotus* using morphology, it is important to examine characters of the male and fourth-instar larva in addition to female characters [18]. Furthermore, until 2012 [25], the only morphological feature that had been proposed to differentiate females of *An. pholidotus* from *An. lepidotus* was the size of the scales on proximal tergites, being moderately wide or broad for *An. lepidotus* and predominantly narrow to moderately wide for *An. pholidotus* [18]. This characteristic was shown vague and difficult to interpret. In fact, in other countries of South America also, such as Colombia, molecular analyses showed that the species previously believed to be *An. lepidotus* corresponded to *An. pholidotus* [47].

Chromosomal banding pattern of the ovarian polytene chromosomes had already suggested that there are three putative species under the name *An. cruzii*, termed as A, B, and C [26,55]. More recently, studies employing DNA sequences from nuclear genes have corroborated this hypothesis [56,57,58,59]. Our analysis, using DNA sequences from mitochondrial genes, also supported the hypotheses of *An. cruzii* representing a complex of morphologically similar species. The sequences employed in this study that were generated from specimens identified as *An. cruzii* were separated in accordance to their geographical origin. Sequence with the GenBank code KU551284 was recovered in a clade that is sister to *An. laneanus* and yet separate from other specimens of *An. cruzii.* This KU551284 sequence was obtained from a mosquito collected in the Parque da Serra da Cantareira, situated in the mountain range north of the municipality of São Paulo [27]. The KU551285 sequence was also sorted separately from all the other *cruzii* sequences and was obtained from a mosquito collected in Itatiaia National Park, in the Mantiqueira mountain range, between the states of Rio de Janeiro and Minas Gerais [27]. All the other *An. cruzii* sequences were grouped in a branch composed by sequences from mosquitoes collected only in the Serra do Mar region, a mountainous chain that extends by approximately 1500 km along the coast, from Rio de Janeiro state to the north of Santa Catarina state. Indeed, at least two genetically distinct *An. cruzii* lineages have been shown to occur in the mountains covered by the Atlantic Forest in South-East Brazil [60]. Here, through another methodology, we confirmed these two populations and showed that a third, coming from another mountain range, the Serra da Cantareira, also separates from the other two in the phylogenetic trees. These three populations of *An. cruzii* were consistently separated regardless of the methodology used (analysis of mitochondrial or nuclear genes). Moreover, in our phylogenetic trees, the addition of another COI gene region as well as another mitochondrial gene (*ND4*) has not changed the topology of the tree, showing that the most important information is contained in the barcoding region, confirming that the COI gene is more useful for identifying sibling and cryptic species, as has been demonstrated [51].

We also demonstrated that mosquitoes morphologically identified as *An. bellator* (CA1, B1, B2, and B3) are likely *An. cruzii*. In fact, adult females of these species are morphologically similar and identification can be problematic when the adults were damaged during field collections [18]. In addition to the placement of these mosquitoes on the phylogenetic trees, their grouping based on the geographical region of the field collections confirms that *An. cruzii* is a species complex.

## 5. Conclusions

The analysis of genetic differentiation of *COI* and *ND4* genes indicated that there are at least two *Kerteszia* species occurring in Peru: *An. boliviensis* and *An. pholidotus.* The occurrence of these species in Peru is herein registered for the first-time, using *COI* DNA sequences. Moreover, we confirmed the occurrence of two subpopulations of *An. cruzii* in the Atlantic rainforest and showed a third population in the mountain range of the Parque da Serra da Cantareira. This population is likely a distinct species that clustered separately from the remaining two lineages of *An. cruzii* in the phylogenetic trees. One lineage encompasses specimens from Serra do Mar on the Atlantic coast, and the second lineage is formed by *An. cruzii* from Serra da Mantiqueira. Besides their placement in the phylogenetic tree, the grouping of *An. cruzii* sequences reflects the geographical origin of the adults (Serra da Cantareira, Serra do Mar, and Serra da Mantiqueira). Thus, these findings add additional support for the *An. cruzii* species complex.

## Figures and Tables

**Table 1:** Main features of the specimens from Peru used in this study and GenBank accession numbers.

Mosquito ID	Origin	Collection Year	Species (According to Morphological Identification)	Species (According to Phylogeny Analysis)	COI * (COIF/COIR Primers)	ND4 *	COI * (LCO/HCO Primers)
1A	Miraflores, Yanatile, Calca, Cusco, Peru	2007	*Anopheles (Kerteszia) boliviensis*	*Anopheles (Kerteszia) boliviensis*	MH589387	MH560305	--
1B	Miraflores, Yanatile, Calca, Cusco, Peru	2007	*Anopheles (Kerteszia) boliviensis*	*Anopheles (Kerteszia) boliviensis*	MH589388	MH560306	MH589377
2A	Corimayo, Yanatile, Calca, Cusco, Peru	2008	*Anopheles (Kerteszia) boliviensis*	*Anopheles (Kerteszia) boliviensis*	MH589389	MH560307	--
2B	Corimayo, Yanatile, Calca, Cusco, Peru	2008	*Anopheles (Kerteszia) boliviensis*	*Anopheles (Kerteszia) boliviensis*	MH589390	MH560308	MH589378
3A	Colca, Yanatile, Calca, Cusco, Peru	2008	*Anopheles (Kerteszia) boliviensis*	*Anopheles (Kerteszia) boliviensis*	MH589391	MH560309	MH589379
3B	Colca, Yanatile Calca, Cusco, Peru	2008	*Anopheles (Kerteszia) boliviensis*	*Anopheles (Kerteszia) boliviensis*	MH589392	MH560310	MH589380
4A	Miraflores, Yanatile, Calca, Cusco, Peru	2008	*Anopheles (Kerteszia) cruzii*	*Anopheles (Kerteszia) boliviensis*	MH589393	MH560311	MH589381
4B	Miraflores, Yanatile, Calca, Cusco, Peru	2008	*Anopheles (Kerteszia) cruzii*	*Anopheles (Kerteszia) boliviensis*	MH589394	MH563012	MH589382
5A	Colca, Yanatile, Calca, Cusco, Peru	2008	*Anopheles (Kerteszia) lepidotus*	*Anopheles (Kerteszia) pholidotus*	MH589395	MH563013	--
5B	Colca, Yanatile, Calca, Cusco, Peru	2008	*Anopheles (Kerteszia) lepidotus*	*Anopheles (Kerteszia) pholidotus*	MH589396	MH560314	--
6A	Miraflores, Yanatile, Calca, Cusco, Peru	2008	*Anopheles (Kerteszia) lepidotus*	*Anopheles (Kerteszia) boliviensis*	MH589397	MH560315	MH589383
6B	Miraflores, Yanatile, Calca, Cusco, Peru	2008	*Anopheles (Kerteszia) lepidotus*	*Anopheles (Kerteszia) pholidotus*	MH589398	MH563016	MH589384
LAN	Selva Alegre, Alto Inambari, Sandia, Puno, Peru	2000	*Anopheles (Kerteszia) laneanus*	*Anopheles (Kerteszia) boliviensis*	MH589409	---	MH589367

* COI, cytochrome c oxidase subunit I gene; ND4, NADH dehydrogenase subunit 4 gene.

**Table 2:** Main features of the specimens from Brazil used in this study and GenBank accession numbers.

Mosquito ID	Origin	Collection Year	Species (According Morphological Identification)	Species (According Phylogeny Analysis)	COI * (COIF/COIR Primers)	ND4 *	COI * (LCO/HCO Primers)
CA1	Caraguatatuba, SP, Brazil	1946	*Anopheles (Kerteszia) bellator*	*Anopheles (Kerteszia) cruzii*	MH589406	MH560328	MH589361
B1	Marsilac, São Paulo, SP, Brazil	2009	*Anopheles (Kerteszia) bellator*	*Anopheles (Kerteszia) cruzii*	--	MH560317	MH589362
B2	Marsilac, São Paulo, SP, Brazil	2009	*Anopheles (Kerteszia) bellator*	*Anopheles (Kerteszia) cruzii*	MH589399	MH560318	MH589363
B3	Marsilac, São Paulo, SP, Brazil	2009	*Anopheles (Kerteszia) bellator*	*Anopheles (Kerteszia) cruzii*	--	MH560319	MH589364
PAC1	Parelheiros, São Paulo, SP, Brazil	2002	*Anopheles (Kerteszia) cruzii*	*Anopheles (Kerteszia) cruzii*	MH589412	MH560333	MH589352
PAC2	Parelheiros, São Paulo, SP, Brazil	2002	*Anopheles (Kerteszia) cruzii*	*Anopheles (Kerteszia) cruzii*	MH589414	MH560335	MH589353
PAC3	Parelheiros, São Paulo, SP, Brazil	2002	*Anopheles (Kerteszia) cruzii*	*Anopheles (Kerteszia) cruzii*	MH589415	MH560336	MH589354
PAC4	Parelheiros, São Paulo, SP, Brazil	2002	*Anopheles (Kerteszia) cruzii*	*Anopheles (Kerteszia) cruzii*	MH589416	MH560337	MH589355
PAC5	Parelheiros, São Paulo, SP, Brazil	2002	*Anopheles (Kerteszia) cruzii*	*Anopheles (Kerteszia) cruzii*	MH589417	MH560338	MH589356
PAC6	Parelheiros, São Paulo, SP, Brazil	2002	*Anopheles (Kerteszia) cruzii*	*Anopheles (Kerteszia) cruzii*	MH589418	--	MH589357
PAC7	Parelheiros, São Paulo, SP, Brazil	2002	*Anopheles (Kerteszia) cruzii*	*Anopheles (Kerteszia) cruzii*	MH589419	MH560339	MH589358
PAC8	Parelheiros, São Paulo, SP, Brazil	2002	*Anopheles (Kerteszia) cruzii*	*Anopheles (Kerteszia) cruzii*	MH589420	MH560340	MH589359
PAC9	Parelheiros, São Paulo, SP, Brazil	2002	*Anopheles (Kerteszia) cruzii*	*Anopheles (Kerteszia) cruzii*	MH589421	MH560341	MH589360
PAC10X	Parelheiros, São Paulo, SP, Brazil	2002	*Anopheles (Kerteszia) cruzii*	*Anopheles (Kerteszia) cruzii*	MH589413	MH560334	--
C1	Marsilac, São Paulo, SP, Brazil	2009	*Anopheles (Kerteszia) cruzii*	*Anopheles (Kerteszia) cruzii*	--	MH560320	MH589369
C2	Marsilac, São Paulo, SP, Brazil	2009	*Anopheles (Kerteszia) cruzii*	*Anopheles (Kerteszia) cruzii*	MH589400	MH560321	MH589370
C3	Marsilac, São Paulo, SP, Brazil	2009	*Anopheles (Kerteszia) cruzii*	*Anopheles (Kerteszia) cruzii*	--	MH560322	MH589371
C4	Marsilac, São Paulo, SP, Brazil	2009	*Anopheles (Kerteszia) cruzii*	*Anopheles (Kerteszia) cruzii*	MH589401	MH560323	MH589372
C5	Marsilac, São Paulo, SP, Brazil	2009	*Anopheles (Kerteszia) cruzii*	*Anopheles (Kerteszia) cruzii*	MH589402	MH560324	MH589373
C6	Marsilac, São Paulo, SP, Brazil	2009	*Anopheles (Kerteszia) cruzii*	*Anopheles (Kerteszia) cruzii*	MH589403	MH560325	MH589374
C7	Marsilac, São Paulo, SP, Brazil	2009	*Anopheles (Kerteszia) cruzii*	*Anopheles (Kerteszia) cruzii*	MH589404	MH560326	MH589375
C8	Marsilac, São Paulo, SP, Brazil	2009	*Anopheles (Kerteszia) cruzii*	*Anopheles (Kerteszia) cruzii*	MH589405	MH560327	MH589376
L1	Campos do Jordão, SP, Brazil	1997	*Anopheles (Kerteszia) laneanus*	*Anopheles (Kerteszia) laneanus*	MH589407	MH560329	MH589365
L3	Campos do Jordão, SP, Brazil	1997	*Anopheles (Kerteszia) laneanus*	*Anopheles (Kerteszia) laneanus*	MH589408	MH560330	MH589366
M95	Itanhaém, SP, Brazil	2010	*Anopheles (Kerteszia) cruzii*	*Anopheles (Kerteszia) cruzii*	MH589411	MH560332	MH589386
M83	Itanhaém, SP, Brazil	2010	*Anopheles (Anopheles) maculipes/pseudomaculipes*	*Anopheles (Anopheles) intermedius*	MH589410	MH560331	MH589385
PAN1	Parelheiros, São Paulo, SP, Brazil	2002	*Anopheles (Nyssorhynchus)* sp.	*Anopheles (Nysssorhynchus) strodei*	--	MH560342	MH589368

* COI, cytochrome c oxidase subunit I gene; ND4, NADH dehydrogenase subunit 4 gene.

**Table 3:** GenBank accession numbers of the reference mosquitoes used in the study.

Species	Mosquito ID	Origin	# GenBank
*An. lepidotus*	51917879	Napo/Ecuador	JQ041282 *
*An. lepidotus*	51917894	Napo/Ecuador	JQ041284 *
*An. pholidotus*	51917595	Tachira/Venezuela	JQ041287 *
*An. pholidotus*	51917607	Tachira/Venezuela	JQ041288 *
*An. homunculus*	BA22_33	Camacan, BA/Brazil	JQ291234 *
*An. homunculus*	ES10_2	Santa Teresa, ES/Brazil	JQ291235 *
*An. homunculus*	RS20_21	Maquiné, RS/Brazil	JQ291236 *
*An. homunculus*	RS20_33	Maquiné, RS/Brazil	JQ291237 *
*An. homunculus*	RS30_56	Maquiné, RS/Brazil	JQ291238 *
*An. homunculus*	SP23_1	Cananéia, SP/Brazil	JQ291240 *
*An. homunculus*	ST26	Cananéia, SP/Brazil	JQ291242 *
*An. lepidotus*	CCDB-10764-A9	Orellana/Ecuador	KF670991 *
*An. cruzii*	???	Cananéia/SP	KJ701506
*An. cruzii*	104	Tapiraí, SP/Brazil	KM507293 *
*An. cruzii*	110	Tapiraí, SP/Brazil	KM507294 *
*An. cruzii*	112	Tapiraí, SP/Brazil	KM507295 *
*An. cruzii*	127	Tapiraí, SP/Brazil	KM507296 *
*An. cruzii*	192	Tapiraí, SP/Brazil	KM507297 *
*An. cruzii*	198	Tapiraí, SP/Brazil	KM507298 *
*An. cruzii*	199	Tapiraí, SP/Brazil	KM507299 *
*An. cruzii*	200	Tapiraí, SP/Brazil	KM507300 *
*An. cruzii*	217	Tapiraí, SP/Brazil	KM507301 *
*An. cruzii*	240	Tapiraí, SP/Brazil	KM507302 *
*An. cruzii*	241	Tapiraí, SP/Brazil	KM507303 *
*An. cruzii*	242	Tapiraí, SP/Brazil	KM507304 *
*An. cruzii*	255	Tapiraí, SP/Brazil	KM507305 *
*An. cruzii*	257	Tapiraí, SP/Brazil	KM507306 *
*An. cruzii*	258	Tapiraí, SP/Brazil	KM507307 *
*An. cruzii*	259	Tapiraí, SP/Brazil	KM507308 *
*An. cruzii*	260	Tapiraí, SP/Brazil	KM507309 *
*An. cruzii*	261	Tapiraí, SP/Brazil	KM507310 *
*An. cruzii*	262	Tapiraí, SP/Brazil	KM507311 *
*An. cruzii*	267	Tapiraí, SP/Brazil	KM507312 *
*An. cruzii*	269	Tapiraí, SP/Brazil	KM507314 *
*An. homunculus*	BA22_32	Camacan/BA	KU551283
*An. cruzii*	PEC_2_7	São Paulo/SP	KU551284
*An. cruzii*	RJ03_2	Itatiaia/RJ	KU551285
*An. cruzii*	RS32_11_7	Maquiné/RS	KU551286
*An. bellator*	SP24_3_1	Cananéia/SP	KU551287
*An. laneanus*	SP52-103	Pindamonhangaba/SP	KU551288
*An. cruzii*	ST16	Cananéia/SP	KU551289
*An. neivai*	MB10165	Petit-Saut/French Guyana	MF172273 *
*An. neivai*	MB10252	Petit-Saut/French Guyana	MF172274 *
*An. neivai*	MB10253	Petit-Saut/French Guyana	MF172275 *
*An. neivai*	MB10254	Petit-Saut/French Guyana	MF172276 *
*An. homunculus*	BA22_31	Camacan/BA	MF381605
*An. laneanus*	SP52-17	Pindamonhangaba/SP	MF381613
*An. homunculus*	RS30_158	Maquiné/RS	MF381697
*An. (An.) intermedius*	SP02_8_1	Pariquera-Açú/SP	MF381700
*An. (Nyr.) strodei*	SP104_18_1	Pindamonhangaba/SP	MF381724 *
*An. homunculus*	ST19	Cananéia/SP	MF381729

* Only for COI barcoding region analysis. **#** GenBank accession number (https://www.ncbi.nlm.nih.gov/genbank/).
